# Severe pulmonary hypertension–interstitial lung disease presenting as right ventricular failure: stabilisation with intravenous prostacyclin and maintenance with inhaled prostacyclin

**DOI:** 10.1183/23120541.00659-2023

**Published:** 2024-01-29

**Authors:** Raj Parikh, Alysse Thomas, Aldo Sharofi, Niala Moallem, Garrett Fiscus, Harrison W. Farber

**Affiliations:** 1 Division of Pulmonary, Critical Care and Sleep, Hartford Hospital, Hartford, CT, USA; 2 A.T. Still University Osteopathic Medical School, Mesa, AZ, USA; 3 University of Connecticut, Storrs, CT, USA; 4 Department of Internal Medicine, University of Connecticut School of Medicine, Farmington, CT, USA; 5 Division of Pulmonary, Critical Care and Sleep, University of Connecticut School of Medicine, Farmington, CT, USA; 6 Division of Pulmonary, Sleep and Critical Care Medicine, Tufts Medical Center, Boston, MA, USA

## Abstract

**Background:**

Pulmonary hypertension (PH) leads to increased morbidity and mortality in interstitial lung disease (ILD). While the INCREASE trial highlighted the use of inhaled prostacyclin in PH-ILD patients, such therapy may be inadequate when right ventricular failure (RVF) is also present. In this study, we report the use of intravenous prostacyclin in three PH-ILD patients to stabilise right ventricular (RV) function, with a subsequent transition to maintenance therapy with inhaled prostacyclin.

**Methods:**

We evaluated three consecutive PH-ILD patients with RVF. RV afterload and pulmonary vascular resistance (PVR) were treated with intravenous prostacyclin during the induction phase of the therapy. Patients transitioned from intravenous prostacyclin to the maintenance phase of the treatment with inhaled prostacyclin once three transition criteria were met: cardiac index (CI) >2 L·min^−1^·m^−2^, PVR <7 Wood units (WU) and tricuspid annular plane systolic excursion (TAPSE) change >1 mm or TAPSE >1.6 cm.

**Results:**

Pre-treatment parameters for the three patients were a mean PVR of 14.3 WU, a mean Fick CI of 1.8 L·min^−1^·m^−2^ and a mean TAPSE of 1.4 cm. The average intravenous prostacyclin dose at the time of transition to maintenance therapy was 20.7 ng·kg^−1^·m^−2^ of treprostinil. At 3-months follow-up, the mean PVR was 6.3 WU, Fick CI 2.2 L·min^−1^·m^−2^ and TAPSE 1.7 cm.

**Conclusion:**

This case series of three PH-ILD patients with RVF introduces the concept of an initial intravenous prostacyclin induction phase, followed by a transition to maintenance therapy with inhaled prostacyclin. Further development of this treatment algorithm with a refinement of the transition criteria, potential testing in a clinical trial and a longer-term follow-up period is warranted to improve the outcomes of advanced PH-ILD patients with concomitant RVF.

## Tweetable abstract

**Treatment algorithm for PH-ILD patients with concomitant right ventricular failure** https://bit.ly/3FTkRiW

## Background

Pulmonary hypertension (PH) worsens the prognosis of interstitial lung disease (ILD) and results in a deteriorating functional status, an increasing need for supplemental oxygen and leading to poorer outcomes [1–5]. The prevalence of PH increases with the progression of lung disease; more than 60% of end-stage ILD may have concomitant PH [6–9].

The clinical utility of PH-specific therapy in PH-ILD patients was demonstrated by the INCREASE trial, in which treatment with inhaled treprostinil led to an improvement in 6-min walk distance [10, 11]. However, many ILD patients present with more severe PH than those enrolled in the INCREASE trial. As such, two issues arise, as follows: 1) how to suspect and diagnose PH earlier in the course of the disease and 2) what actions to take in those patients who present with right ventricular failure (RVF). The first issue can be addressed through use of the recently developed PH-ILD detection tool [12, 13]. The second issue is somewhat more challenging in that RVF can further complicate the management similar to what occurs in decompensated World Health Organization (WHO) Group 1 pulmonary arterial hypertension (PAH) [12–17]. In such patients, parenteral prostacyclin therapy with treprostinil or epoprostenol may be considered [14, 15]. While parenteral prostacyclins have well-established benefits in WHO Group 1 PAH, similar results are not as evident in PH-ILD because such treatment has the potential to exacerbate hypoxaemia by causing ventilation/perfusion (*V*ʹ/*Q*ʹ) mismatch in the setting of parenchymal lung disease [14, 15, 18].

In PH-ILD patients with concomitant RVF, the initial goal of therapy should focus on stabilisation and improvement of RV function by rapidly decreasing the pulmonary vascular resistance (PVR), similar to the management of RVF in PAH [19]. Once the RV function has improved sufficiently, there is potential for long-term management using inhaled prostacyclin therapy, akin to the approach employed in the INCREASE trial [10, 11]. Here, we report the use of intravenous prostacyclin therapy in three PH-ILD patients to achieve stabilisation and improvement in RV function followed by a transition to maintenance inhaled prostacyclin therapy.

## Methods

We performed a retrospective analysis of three consecutive PH-ILD patients who presented with concomitant acute RVF and were admitted to a single tertiary academic centre. The diagnosis of ILD was confirmed by diffuse parenchymal lung disease on chest computed tomography. The diagnosis of pre-capillary PH was confirmed by right heart catheterisation (RHC) with a mean pulmonary pressure (mPAP) ≥20 mmHg, pulmonary capillary wedge pressure ≤15 mmHg and PVR >3 Wood units (WU) [20]. Other causes of pre-capillary PH, including chronic thromboembolic pulmonary hypertension and connective tissue disease, were excluded.

Acute RVF was defined by transthoracic echocardiogram (TTE) and invasive haemodynamic data; criteria included RV dysfunction on echocardiogram (tricuspid annular plane systolic excursion (TAPSE) <1.6 cm) as well as an elevated right atrial pressure (RAP) (≥15 mmHg) and a depressed indirect Fick cardiac index (CI) (<2 L·min^−1^·m^−2^) [20–24].

### Induction protocol

During the index admission, all patients were newly diagnosed with PH-ILD and treatment naïve in regards to pulmonary vasodilator therapy. RHC was performed and an indwelling pulmonary artery catheter (PAC) secured in place for continuous haemodynamic monitoring in the intensive care unit (ICU). Patients received therapy for RVF aimed at optimisation of RV pre-load and contractility. This included continuous infusion of high-dose loop diuretics, as well as vasopressors and/or inotropic agents.

**FIGURE 1:**
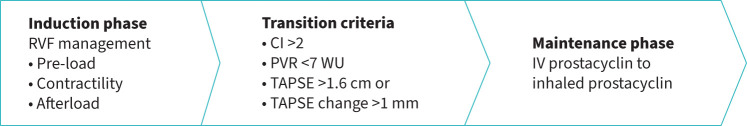
Induction phase, transition criteria and maintenance phase. CI: cardiac index; IV: intravenous; PVR: pulmonary vascular resistance; RVF: right ventricular failure; TAPSE: tricuspid annular plane systolic excursion; WU: Wood unit.

In addition, RVF was treated with parenteral prostacyclin therapy administered *via* a peripherally inserted central catheter line. Uptitration of parenteral prostacyclin and subsequent optimisation of RV afterload, in conjunction with RV pre-load and contractility, was guided by haemodynamics from the PAC. Goals of therapy were not only to achieve a target Fick CI of >2 L·min^−1^·m^−2^ and a PVR <7 WU, but also to demonstrate echocardiographic changes in the RV as measured by a TAPSE improvement by >1 mm or TAPSE >1.6 cm [10, 20, 25–28]. CI was calculated by the Fick principle, where blood sampling was obtained from a systemic artery and the pulmonary artery to calculate the arterial–venous oxygen difference. Patients underwent daily TAPSE assessment by a critical care TTE board-certified physician. The induction phase of advanced prostacyclin administration ended and the transition to the maintenance inhaled prostacyclin phase initiated when all transition criteria were fulfilled (figure 1).

### Transition criteria

As the patient's RV pre-load, contractility and afterload were addressed by diuretics, vasopressors, inotropes and parenteral prostacyclin therapy during the induction phase, daily monitoring with PAC and TTE was performed to evaluate the patient's eligibility to proceed to the transition phase of the management protocol.

Transition criteria included a PAC-derived haemodynamic improvement in CI to >2 L·min^−1^·m^−2^ [20]. In conjunction with CI, a PVR <7 WU was included in the transition criteria; this cut-off was chosen because the patients in the INCREASE trial had a mean PVR of 6.4 WU [10]. Lastly, echocardiographic improvement in TAPSE by >1 mm or TAPSE >1.6 cm was also included [25–28].

When all three criteria (CI, PVR and TAPSE) were fulfilled, patients were considered eligible for transition from the induction portion of the management protocol to the maintenance phase, namely transition from intravenous prostacyclin to inhaled prostacyclin (figure 1).

### Intravenous to inhaled prostacyclin transition

Once the CI, PVR and TAPSE criteria were fulfilled, patients were transitioned from intravenous prostacyclin to inhaled prostacyclin based on prior protocols [28–30]. A conversion value of approximately 1 ng·kg^−1^·m^−2^ of intravenous treprostinil to one breath of inhaled treprostinil was utilised [28–30]. The transition took place in the ICU over 24 h. None of the patients suffered any major adverse events or side-effects during this transition.

## Results

### Baseline clinical characteristics

The baseline clinical characteristics of the three PH-ILD patients are detailed in table 1. The mean age was 75 years and two of the three patients were male (66.7%). Two patients had combined pulmonary fibrosis with emphysema (CPFE) and one patient had idiopathic pulmonary fibrosis; none of the patients had received antifibrotic therapy. All three patients required chronic supplemental oxygen therapy at the time of admission. None of the patients had been evaluated for PH as an outpatient prior to admission. Mean pro-brain natriuretic peptide (pro-BNP) at the time of admission was 15 753 pg·mL^−1^ and all three patients exhibited signs of right heart failure on physical exam (table 2).

**TABLE 1:** Clinical characteristics of pulmonary hypertension–interstitial lung disease (ILD) patients with right ventricular failure at time of admission

**Patient ID**	**Age**	**Ethnicity**	**Sex**	**Prior smoker**	**ILD type**	**Length of ILD diagnosis (months)**	**Antifibrotic treatment**	**FVC (%)**	***D*_LCO_ (%)**	**Use of supplemental oxygen**
**1**	75	W	F	Yes	CPFE	16	No	53	18	Yes
**2**	77	W	M	No	IPF	7	No	60	30	Yes
**3**	72	W	M	Yes	CPFE	8	No	–	–	Yes

–: No data available; CPFE: combined pulmonary fibrosis with emphysema; *D*_LCO_: diffusing capacity of the lung for carbon monoxide; F: female; FVC: forced vital capacity; IPF: idiopathic pulmonary fibrosis; M: male; W: white.

**TABLE 2:** Right ventricular failure signs and symptoms at time of admission

**Patient ID**	**JVD present**	**LE oedema present**	**Pro-BNP (pg·mL^−1^)** **^#^**	**WHO FC**
**1**	Yes	Yes	8647	4
**2**	Yes	Yes	31 501	4
**3**	Yes	Yes	7111	4

**^#^**Normal range pro-brain natriuretic peptide (pro-BNP): <125 pg·mL^−1^. JVD: jugular venous distension; LE: lower extremity; WHO FC: World Health Organization functional class.

### Baseline RHC and TTE prior to PH therapy

The invasive haemodynamics at the time of initial, treatment-naïve RHC are detailed in table 3. The mPAP was 57 mmHg, mean PVR was 14.3 WU and the mean Fick CI was 1.8 L·min^−1^·m^−2^. Pre-treatment mean TAPSE obtained during index hospitalisation TTE was 1.4 cm.

**TABLE 3:** Right ventricular failure and transthoracic echocardiogram data prior to therapy initiation

**Patient ID**	**RAP** **(mmHg)**	**mPAP (mmHg)**	**PAWP (mmHg)**	**PVR (WU)**	**Fick CI (L·min^−1^·m^−2^)**	**TAPSE (cm)**	**IVS flattening**
**1**	18	62	10	17	1.7	1.4	Yes
**2**	23	58	11	16	1.8	1.4	Yes
**3**	20	51	14	10	1.8	1.5	Yes

CI: cardiac index; IVS: interventricular septum; mPAP: mean pulmonary artery pressure; PAWP: pulmonary artery wedge pressure; PVR: pulmonary vascular resistance; RAP: right atrial pressure; TAPSE: tricuspid annular plane systolic excursion; WU: Wood unit.

### Index hospitalisation characteristics

The patients had an average ICU length of stay (LOS) of 24.7 days (table 4). All three patients initially required haemodynamic support with either an inotrope or a vasopressor. Intravenous prostacyclin was initiated within 4 days of admission. The average dose of intravenous prostacyclin required to fulfil criteria for transition to maintenance therapy was 20.7 ng·kg^−1^·m^−2^ treprostinil (table 5). Post-induction data including PVR, Fick CI and TAPSE are presented in table 5.

**TABLE 4:** Index hospitalisation

**Patient ID**	**ICU LOS**	**Vasopressors (max dose)**	**Inotrope (max dose)**	**Continuous-infusion diuretic (max dose)**	**Intravenous prostacyclin (days)**
**1**	21	Norepinephrine (8 μg·min^−1^) Vasopressin (0.03 units·min^−1^)	Dobutamine (2.5 μg·kg^−1^·min^−1^)	Furosemide (20 mL·h^−1^)	17
**2**	29	Norepinephrine (13 μg·min^−1^) Vasopressin (0.03 units·min^−1^)	Dobutamine (2.5 μg·kg^−1^·min^−1^)	Furosemide (25 mL·h^−1^)	24
**3**	24	Norepinephrine (12 μg·min^−1^) Vasopressin (0.03 units·min^−1^)	Dobutamine (2.5 μg·kg^−1^·min^−1^)	Furosemide (25 mL·h^−1^)	20

ICU LOS: intensive care unit length of stay; max: maximum.

**TABLE 5:** Right ventricular failure and transthoracic echocardiogram measurements at time of transition from intravenous to inhaled prostacyclin

**Patient ID**	**Intravenous prostacyclin dose (ng·kg^−1^·min^−1^)**	**Supplemental O_2_ needs compared to baseline**	**RAP (mmHg)**	**mPAP (mmHg)**	**PAWP (mmHg)**	**PVR (WU)**	**Fick CI (L·min^−1^·m^−2^)**	**TAPSE (cm)**	**Inhaled prostacyclin dose at discharge (breaths four times daily)**
**1**	24	Unchanged	10	40	14	6.9	2.1	1.6	24
**2**	18	Decreased	9	37	13	6.6	2.3	1.6	18
**3**	20	Decreased	8	44	14	6.3	2.2	1.7	18

CI: cardiac index; mPAP: mean pulmonary artery pressure; PAWP: pulmonary artery wedge pressure; PVR: pulmonary vascular resistance; RAP: right atrial pressure; TAPSE: tricuspid annular plane systolic excursion; WU: Wood unit.

### Longitudinal outcomes in response to maintenance therapy

3 months after index hospitalisation, patients underwent surveillance TTE and RHC (table 6); mean PVR was 6.3 WU, mean Fick CI was 2.2 L·min^−1^·m^−2^ and mean TAPSE was 1.7 cm. Apart from inhaled prostacyclin, the patients were not receiving any other pulmonary vasodilator therapy at the 3-month assessment. None of the patients were re-admitted during this follow-up period and the WHO functional class improved from a mean of 4 prior to induction therapy to 3 at the follow-up assessment.

**TABLE 6:** Status 3 months after discharge

**Patient ID**	**Inhaled prostacyclin dose (breaths four times daily)**	**RAP (mmHg)**	**mPAP (mmHg)**	**PAWP (mmHg)**	**PVR (WU)**	**Fick CI (L·min^−1^·m^−2^)**	**TAPSE (cm)**	**IVS flattening**
**1** **^#^**	18	6	38	12	6.6	2.2	1.6	Yes
**2**	18	7	34	11	6.1	2.3	1.7	No
**3**	18	7	43	14	6.2	2.2	1.7	Yes

^#^: patient 1 was discharged on a dose of 24 breaths four times daily but titrated down to 18 breaths four times daily due to side-effects of headache and cough. CI: cardiac index; IVS: interventricular septum; mPAP: mean pulmonary artery pressure; PAWP: pulmonary artery wedge pressure; PVR: pulmonary vascular resistance; RAP: right atrial pressure; TAPSE: tricuspid annular plane systolic excursion; WU: Wood unit.

## Discussion

WHO Group 1 PAH patients with evidence of RV dysfunction resulting in depressed CI, elevated RAP and significantly elevated PVR are usually categorised as high risk when assessed by various PAH risk calculators [20, 31, 32]. In such patients, parenteral prostacyclin therapy is indicated [19, 20, 31, 32]. A similar concept might be extrapolated to other forms of pre-capillary PH with concomitant RVF, including PH-ILD [33]. However, long-term parenteral prostacyclin therapy in PH-ILD is not standard of practice and *V*ʹ/*Q*ʹ mismatch in the setting of parenchymal lung disease may occur [14, 15, 18, 20]. As such, administration of parenteral prostacyclin therapy in PH-ILD patients with concomitant RV failure should only be performed in a closely monitored setting by an experienced PH specialist [15]. None of the patients in this cohort had increasing supplemental oxygen requirements (table 5).

Our case series of three PH-ILD patients with RVF lays the groundwork for a potential treatment algorithm for these complex patients, including utilisation of intravenous prostacyclin during an induction phase with RHC and TTE-defined transition criteria leading to long-term maintenance therapy with inhaled prostacyclin.

### Mirroring the approach with intravenous to oral treprostinil

The preliminary data from the recent EXPEDITE study of PAH patients created a protocol for transition from parenteral prostacyclin therapy to a less invasive route of maintenance administration after an induction period [34]. While the primary goal of the EXPEDITE study was to allow patients to acclimate to the side-effect profile of oral treprostinil by administering the drug intravenously during the initial period, similar principles could potentially apply to PH-ILD patients who have developed RVF. The initiation of parenteral prostacyclin therapy in these patients may provide benefits in terms of stabilising and improving RV function. Once this is achieved, patients could transition to inhaled prostacyclin therapy, which is not only an approved therapeutic option in this sub-population of pre-capillary PH, but is also less invasive, more patient-friendly and has a decreased likelihood of causing adverse events such as *V*ʹ/*Q*ʹ mismatch [10, 11, 15, 20].

### What about chronic RV disease?

This approach of managing severe PH associated with acute RVF with an induction phase followed by maintenance therapy is novel; while it has its merits, such an approach may not apply to patients who have had longstanding RV dysfunction. These individuals may no longer have an adaptable RV that is capable of stabilising and improving even with parenteral prostacyclin therapy [35]. To wit, the three patients in this report all presented with undiagnosed PH and acute RVF.

### Choosing the transition criteria metrics

The justification for the RHC- and TTE-derived transition criteria of CI, PVR and TAPSE was based on prior evidence of each metric playing a significant role in the risk stratification of WHO Group 1 PAH patients, as well as data obtained from the INCREASE trial [10, 20, 31, 32]. However, as this treatment algorithm is incorporated in the management of PH-ILD patients with RVF, we will be able to further refine the transition criteria metrics. As a result, the transition criteria will be tailored and adjusted with increasing experience and long-term follow-up in these patients; cut-offs may change, additional metrics added and/or current parameters removed.

### Effect on resource utilisation: LOS and readmissions

Another obvious issue with this approach to therapy, *i.e.* an induction phase with advanced prostacyclin therapy followed by a transition to inhaled prostacyclin in a closely monitored environment, is the prolonged hospitalisation required. In our case series, the ICU LOS alone was over 24 days. All three patients were suffering from significant deconditioning and required extensive rehabilitation while working towards a safe discharge. Nevertheless, while the ICU LOS may be significant during this induction phase, the long-term benefit of stabilising and improving RF function could, in contrast, prevent such patients from recurrent admissions for RVF, volume overload and acute on chronic hypoxic respiratory failure. In this case series, none of the patients were readmitted to the hospital within 3 months of discharge.

### Limitations and strengths

There are several limitations to this study, as follows: 1) it is a small case series of three patients without substantial longitudinal data beyond a 3-month period and 2) confounding factors, such as inotrope- and vasopressor-assisted diuresis and its beneficial effects on invasive haemodynamics and subsequent improvement in PVR, are unknown. The primary strength of the study is that it introduces a novel treatment algorithm into the management of PH-ILD patients who present with a severe RVF phenotype.

### Conclusion

In this case series of three PH-ILD patients with RVF, we performed an induction phase of therapy with intravenous prostacyclin using echocardiographic and invasive haemodynamic monitoring followed by a transition to maintenance therapy with inhaled prostacyclin prior to discharge. Further development of this treatment algorithm, potential testing in a clinical trial and a longer follow-up is warranted to improve the outcomes of patients with advanced PH-ILD and concomitant RVF.
